# Multi-view emotional expressions dataset using 2D pose estimation

**DOI:** 10.1038/s41597-023-02551-y

**Published:** 2023-09-22

**Authors:** Mingming Zhang, Yanan Zhou, Xinye Xu, Ziwei Ren, Yihan Zhang, Shenglan Liu, Wenbo Luo

**Affiliations:** 1 Research Center of Brain and Cognitive Neuroscience, Liaoning Normal University, Dalian, 116029 Liaoning China; 2 Key Laboratory of Brain and Cognitive Neuroscience, Liaoning Province, Dalian, 116029 China; 3 School of Innovation and Entrepreneurship, Dalian University of Technology, Dalian, 116024 Liaoning China; 4 Faculty of Electronic Information and Electrical Engineering, Dalian University of Technology, Dalian, 116024 Liaoning China

**Keywords:** Psychology, Computer science, Human behaviour

## Abstract

Human body expressions convey emotional shifts and intentions of action and, in some cases, are even more effective than other emotion models. Despite many datasets of body expressions incorporating motion capture available, there is a lack of more widely distributed datasets regarding naturalized body expressions based on the 2D video. In this paper, therefore, we report the multi-view emotional expressions dataset (MEED) using 2D pose estimation. Twenty-two actors presented six emotional (anger, disgust, fear, happiness, sadness, surprise) and neutral body movements from three viewpoints (left, front, right). A total of 4102 videos were captured. The MEED consists of the corresponding pose estimation results (i.e., 397,809 PNG files and 397,809 JSON files). The size of MEED exceeds 150 GB. We believe this dataset will benefit the research in various fields, including affective computing, human-computer interaction, social neuroscience, and psychiatry.

## Background & Summary

It is widely accepted that emotion is communicated via multiple models involving both verbal and non-verbal aspects, such as tone, eye movement, facial expression, and body language. Recent studies have demonstrated that body movements can effectively reflect changes in affective state^1^, even among primates^2^. People pay more attention to body expressions than facial expressions or voices when dealing with affective states such as information in high intensity^3^, perceptual ambiguity conditions^4^, or when information from these channels is incongruent^5,6^. As increasing psychological studies indicated the significant role of body movement in transmitting information and emotional states^7–9^, artificial intelligence for emotion recognition is changing from facial expression system^10^ or body expression system^11,12^ to a multi-channel information combination^13^.

Various domains of studies on body parts movement cover gait analysis^14^, body posture analysis, and gesture analysis. One focus of body movement is kinematic information of body movement such as velocity, acceleration, trajectory, and postures, which cannot be accurately and effectively represented by static pictures or verbal descriptions. In recent decades, motion capture technology has made it possible to precisely capture and analyze the kinematic data of each joint^15–18^. A variety types of stimulus sets have emerged, including point-light displays^19,20^, video clips^21^, images^22^, or virtual agents^14,23,24^. The study of body movement has gradually shifted from concepted research to data-based quantitative research.

However, kinematic information from 2D video is also essential for studying emotional body movements. It is not customary for individuals to equip themselves with sensors, as is commonly done in laboratory settings. Fortunately, many pose estimation projects, such as AlphaPose^25^, Pose Tensorflow^26,27^, OpenPose^28^, and Deeplabcut^29,30^, use machine learning to estimate the posture of persons or animals in videos or pictures and obtain various data, such as the coordinates of joints. They have been applied in some studies in the field of social neuroscience^31–35^. For example, de Gelder and Poyo Solanas proposed the radically distributed model^36^, which suggests an additional mid-level feature analysis between low-level feature and high-level concept analyses. The mid-level features – kinematic features (e.g., velocity, acceleration, vertical movement) and postural features (e.g., limb angle, limb contraction, symmetry, surface, shoulder ratio) – have a specific mapping with the brain. Poyo Solanas, Vaessen, and de Gelder found that the extra-striate body area and fusiform body area exhibit more sensitivity towards postural features than kinematic features^37^.

Therefore, we report a larger and standardized dataset with various emotions: the multi-view emotional expressions dataset (MEED). MEED contains 4102 recordings of six emotional (anger, disgust, fear, happiness, sadness, surprise) and neutral body movements from three views (left, front, right). Each recording consists of the frames extracted by OpenPose and the coordinates of pixel space for 25 body joints in each frame. MEED is freely available. We expect to encourage researchers in multiple fields (e.g., affective computing, human-computer interaction, artificial intelligence, social security, and social neuroscience) to fully explore the various features of emotional body movements in daily life. Interdisciplinary research in these fields should also be promoted.

## Methods

### Preparation phase

Twenty-four college students with acting experience from Dalian University of Technology were recruited with appropriate payment. All participants signed an informed consent, knowing that the recordings they performed would be shared publicly. Two actors dropped out, leaving 22 actors (19–24 years old, mean = 20.6 years) included in the MEED. This study was approved by the Human Research Institutional Review Board of Liaoning Normal University and followed the Declaration of Helsinki (1991).

Thirty-five standardized daily event scenarios (five for each emotion and neutral) with high recognition accuracy (82.9% - 100%, mean = 93.4%) were created to guide the actors in the recording phase. The specific content and validation of these scenarios and performances were introduced in our previous work^20,24^.

Three Microsoft Kinect 2.0 cameras, with a resolution of 15 fps, were placed respectively at the front, left, and right of a 1 m × 1 m sized stage, 1.05 m high from the floor, 2.5 m from the center of the stage, and were controlled by a laptop computer (Microsoft Surface Pro 4). More details can be found in our previous work^38^.

### Recording phase

Actors, wearing in black tights, performed six seconds according to the randomly presented scenario, and several performances were selectively repeated to guarantee robustness. Actors were asked to face the center camera, standing naturally with arms hanging down. All three cameras started recording simultaneously after the actor indicated he/she was ready. The recording phase took approximately two hours, during which the actors may rest at any time.

### Pose estimation

**Fig. 1:**
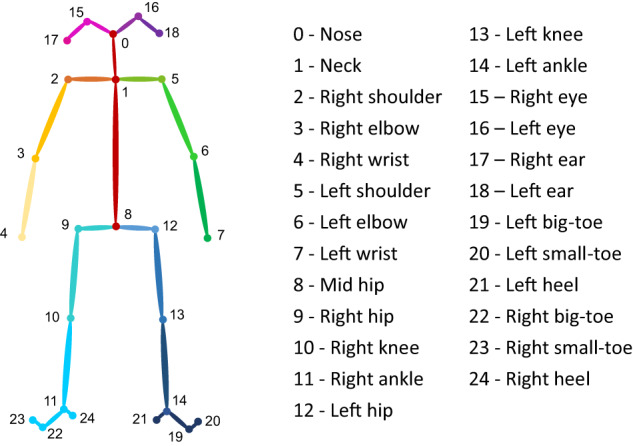
Twenty-five keypoints of the OpenPose software model.

OpenPose (v1.7.0), an advanced, reliable bone-extraction library^28^, uses a convolutional neural network to estimate skeletal joints and coordinates (x, y) of actors’ physical joint points. This dataset is based on 25 points model (i.e., nose, neck, right shoulder, right elbow, right wrist, left shoulder, left elbow, left wrist, mid hip, right hip, right knee, right ankle, left hip, left knee, left ankle, right eye, left eye, right ear, left ear, left big-toe, left small-toe, left heel, right big-toe, right small-toe, and right heel; see Fig. 1).

**Fig. 2:**
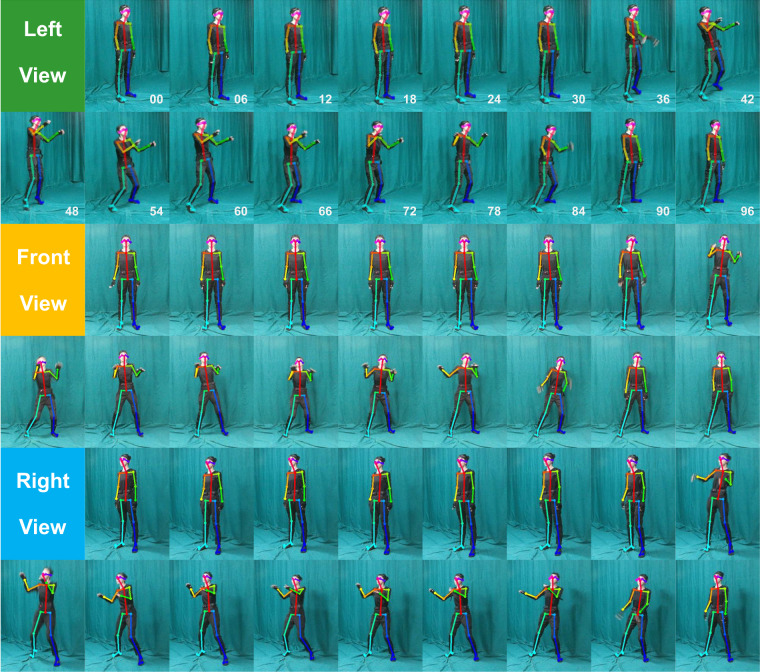
Three examples of multi-view pose estimation. The informed consent to publish the actress’s likeness was obtained.

Each video has 97 image frames (see Fig. 2), except part of which are slightly fewer. The horizontal and vertical coordinates (x, y) of 25 keypoints in the pixel space of each frame for each video, as well as the confidence level for determining joint position, were available through pose estimation. Results from pose estimation have two forms: images and data files of joints position. All image files were composed of image frames, skeletal joints, and 25 keypoints (see Fig. 1). For individual recordings, the information in image files were digitized to the datafile of each frame.

## Data Records

Due to a malfunction in the equipment, there were no frontal view videos recorded of the actor M01. Eventually, 4,162 videos were collected, and the following files were excluded from analysis: one file (left_M04H0V2) was corrupted, two actors (F04 and F13; 54 videos) dropped out, two dance videos (right_F06dance, right_M07dance) were test files, and three videos (front_M03H0V2, front_M06SA0V1, right_M01SA2V1) with severe limb obscuration failed to be estimated by OpenPose (v1.7.0). Therefore, MEED retains 4102 recordings (see Table 1). Among them, 4092 videos contain 97 frames each, while the remaining videos have frames of 96, 77, 95, 87, 75, 98, 98, 98, 68, and 93 respectively for left_F07N3V1, left_F11SA4V1, left_M09SU0V2, front_M09SU0V2, front_M10N4V1, right_F02N4V1, right_F07SA5V1, right_M06h5v1, right_M09N1V2, and right_M09SU0V2. MEED is freely available on Zenodo^39^.

**Table 1:** The number of recordings under all conditions.

Emotions	Views			Total
	Left	Front	Right	
Anger	197	190	196	583
Disgust	208	197	210	615
Fear	216	207	216	639
Happiness	209	204	213	626
Neutral	146	137	146	429
Sadness	198	189	197	584
Surprise	212	202	212	626
Total	1386	1326	1390	4102

All remaining recordings were systematically named as “<view> <actor_id> <emotion> <scenario_id> <version>”, where “view” refers to the point of view, “actor_id” refers to the actor ID, and “emotion” includes anger (A), disgust (D), fear (F), happiness (H), neutral (N), sadness (SA), and surprise (SU). “scenario_id” refers to scenario (1~5) performance and free performance (0), and “version” is the number of repetitions.

The main folder of MEED has 21 actor folders for front view, 22 actor folders for left view, and 22 actor folders for right view. Pose estimation results include PNG files of each frame in individual performance and JSON files about the coordinates of 25 keypoints, named by recording name and the frame number of each frame. MEED totally has 397,809 PNG files and 397,809 JSON files. Moreover, to facilitate the subsequent research, MAT files of coordinates for each recording are available in the corresponding recording folder, and all coordinate files for a single view are summarized in MEED. In the main folder, there is one quality .csv file and one quality .mat file to show the technical validation result of MEED (see Technical Validation section).

## Technical Validation

### Proportion of unrecognized keypoints

The effectiveness of OpenPose in extracting coordinates depends on various factors such as the velocity of the actor’s movement, fps, physical occlusion, etc. A high velocity may cause blurring in some frames and deviations in the position of keypoints.

Additionally, limb occlusion lowers the confidence level for confirming joint positions, and long-term physical occlusion may make subsequent joints unrecognized due to the lack of prior information. Consequently, the coordinates of the unrecognized keypoints in some frames would appear as (0, 0). We consider the proportion of the number of these unrecognized keypoints to the number of all keypoints in all frames of each recording as one of the quality metrics for the 2D pose estimation dataset, called proportion of unrecognized keypoints (PUK), which is defined as

1 $$\begin{array}{c}PUK=\frac{{N}_{\left(0,0\right)}}{{N}_{keypoint}\times {N}_{frame}}\end{array}$$

where *N*_(0,0)_ is the total number of unrecognized keypoints in all frames of each recording, and *N*_*keypoints*_ and *N*_*frame*_ separately refers to 25 keypoints of body pose estimation and total number of frames of each recording.

**Fig. 3:**
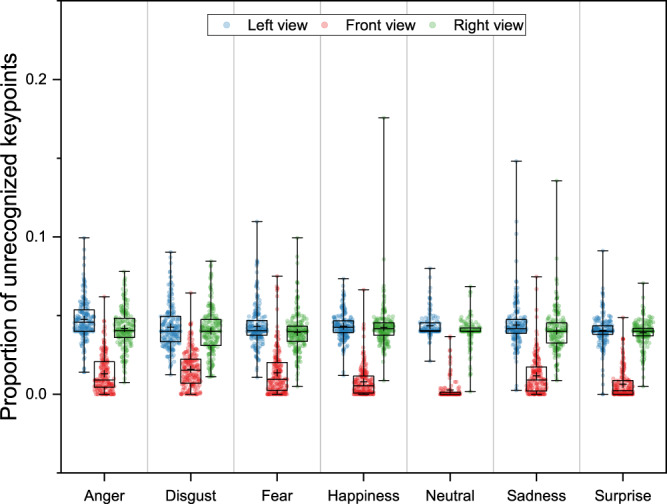
Box plots of the proportion of unrecognized keypoints under all conditions. The cross represents the mean value.

The results showed that the PUK was lowest in the frontal view, with mean values ranging from 0.003 to 0.048 under all conditions (see Table 2 and Fig. 3).

**Table 2:** Mean (and Median) of the proportion of unrecognized keypoints under all conditions.

Emotions	Views		
	Left	Front	Right
Anger	0.048 (0.046)	0.013 (0.009)	0.042 (0.040)
Disgust	0.043 (0.040)	0.016 (0.015)	0.040 (0.040)
Fear	0.043 (0.040)	0.014 (0.009)	0.039 (0.040)
Happiness	0.043 (0.042)	0.008 (0.005)	0.042 (0.042)
Neutral	0.044 (0.040)	0.003 (0.000)	0.040 (0.040)
Sadness	0.044 (0.042)	0.012 (0.009)	0.040 (0.040)
Surprise	0.040 (0.040)	0.006 (0.002)	0.039 (0.040)

### Confidence level

OpenPose uses confidence maps to assess the predicted data, which is created by the annotated keypoints^28^. Every confidence map is a 2D indication of the possibilities that the body part appears at each pixel location. It will generate a possible area of Gaussian distribution, the center of which is the keypoints. The Gaussian center has a maximum confidence of 1. The further away from the center, the lower the confidence is. In other words, each pixel position in the confidence map has a corresponding confidence value. The number of confidence peaks equals the number of people in the picture being predicted. MEED contains only single-person situations, so there is only one peak per confidence map. The ground-truth confidence map generated by the network is to take the maximum confidence value through a non-maximum suppression algorithm.

**Fig. 4:**
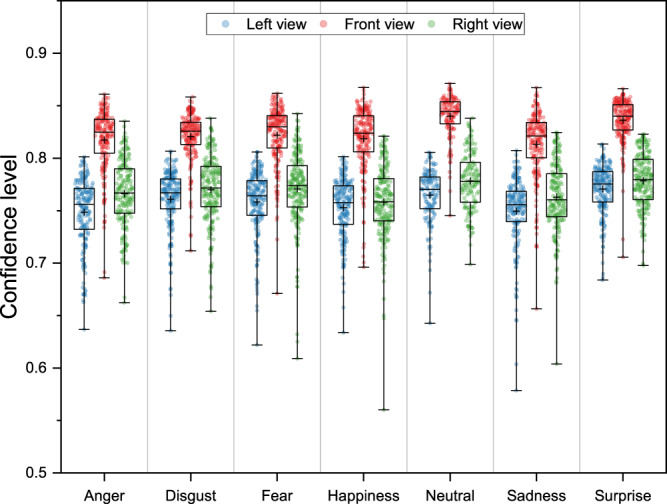
Box plots of the confidence level under all conditions. The cross represents the mean value.

This confidence peak is expressed in the pose estimation results as the confidence level (CL) attached to each keypoint estimation. Therefore, we regard the mean CL of 25 keypoints within each recording as the second quality metric for this dataset, which is defined as

2 $$\begin{array}{c}CL=\frac{{\sum }_{n=1}^{{N}_{frame}\times {N}_{keypoint}}CL}{{N}_{keypoint}\times {N}_{frame}}\end{array}$$

where *N*_*frame*_ and *N*_*keypoint*_ refer to the number of frames in each recording and 25, respectively. To compare the pose estimation in this dataset with the normal level of OpenPose^28^, we analyzed CL in all conditions. Results showed that the CL in the frontal view is the highest. The mean values of CL ranged from 0.748 to 0.840 under all conditions (see Table 3 and Fig. 4). The results of two quality metrics suggest that the pose estimation results are good enough for further analysis.

**Table 3:** Mean (and Median) of confidence level under all conditions.

Emotions	Views		
	Left	Front	Right
Anger	0.748 (0.756)	0.817 (0.825)	0.766 (0.767)
Disgust	0.761 (0.767)	0.821 (0.826)	0.770 (0.771)
Fear	0.758 (0.764)	0.822 (0.830)	0.771 (0.774)
Happiness	0.753 (0.758)	0.818 (0.824)	0.758 (0.758)
Neutral	0.765 (0.770)	0.840 (0.844)	0.778 (0.778)
Sadness	0.750 (0.756)	0.813 (0.821)	0.763 (0.760)
Surprise	0.771 (0.775)	0.836 (0.840)	0.778 (0.779)

### Performance reliability

To ensure that all 22 actors expressed the instructed emotions equally well, that is, the reliability of these performances, we examined the consistency of the objective movement value across all of them. The objective movement of the recordings of frontal view in MEED was quantified using a customized MATLAB code^40,41^, and prior research has been demonstrated that this movement positively correlates with the intensity of emotion and the motion that observers can perceive from human body^20,42,43^. Specifically, if a pixel in two consecutive frames had a luminance change of more than 10 units, it was considered a pixel motion. The objective movement values were depicted by computing the average number of pixel motions in each frame and video, which were then saved in the frontMovement.csv.

We then conducted a reliability analysis of the objective movement value for each emotional and neutral condition across all actors using SPSS 26.0 (https://www.ibm.com/products/spss-statistics). The result showed that the Cronbach alpha coefficient was high under all emotional and neutral conditions (anger = 0.900, disgust = 0.939, fear = 0.919, happiness = 0.875, sadness = 0.929, surprise = 0.927, and neutral = 0.974), suggesting a high reliability of these performances and all actors in MEED express these emotions and scenarios equally.

## Usage Notes

MEED is an open-source library that stores the results of 2D pose estimation with six emotions and neutral expression as well as three views. JSON and MAT files can be easily used by data processing software such as MATLAB (https://ww2.mathworks.cn/en/products/matlab.html), R (https://www.r-project.org), and Python (https://www.python.org). For example, the coordination data can be analyzed using representational similarity analysis^44^ for the association between kinematic features and postural features of body expressions and decision tree classifier^45^ for the relative importance of these features and body parts^46^.

Moreover, the unrecognized coordinates must be fixed if users want to involve them in their analyses. We suggest that users perform interpolation correction, such as linear, polynomial interpolation, and spline interpolation, on the coordinates of individual keypoint in the videos on the time scale as data streams. Given that linear interpolation is limited to the case of non-continuous unrecognized keypoints, we suggest fitting curve instead, such as the Curve Fitting Toolbox in MATLAB (https://ww2.mathworks.cn/products/curvefitting.html) or its built-in functions (spline, makima, pchip). We also recommend using Photoshop (https://www.adobe.com/products/photoshop.html) for PNG correction when necessary.

MEED is applicable in multiple fields, such as the affective computing of body expressions and corresponding brain mechanisms^37,46^ in social neuroscience. Researchers in human-computer interaction, machine learning, sports motion analysis, psychiatry, and social security will also be interested in this dataset. We hope that MEED will be of further assistance to them.

## Data Availability

The MATLAB code for parsing the JSON file and processing the coordinates can be found at 10.5281/zenodo.8185369.
